# Denture-Associated Candidiasis and Mucormycosis in Post-COVID-19 Older Adults Managed Through an Integrated Prosthodontic and Infectious Disease Approach: A Narrative Review

**DOI:** 10.7759/cureus.103448

**Published:** 2026-02-12

**Authors:** Panagiota Chatzidou, Athanasios Stratos, Meira Chint, Athina Niakou, Argirios Pissiotis, Savvas Kamalakidis

**Affiliations:** 1 Prosthodontics, School of Dentistry, Faculty of Health Sciences, Aristotle University of Thessaloniki, Thessaloniki, GRC; 2 General Dentistry, Aristotle University of Thessaloniki, Thessaloniki, GRC

**Keywords:** biofilms, candidiasis, covid-19, dentures, fungal infections, immunosenescence, mucormycosis, older adults, oral health

## Abstract

The COVID-19 pandemic has exposed significant vulnerabilities among older adults, particularly denture wearers, to opportunistic fungal infections, including mucormycosis and oral candidiasis. This narrative review, following PRISMA-ScR (Preferred Reporting Items for Systematic reviews and Meta-Analyses extension for Narrative Reviews) guidelines, collected evidence from 2020 to 2025 to examine the connection between denture use, systemic comorbidities, and fungal complications in elderly individuals after COVID-19. A total of 21 of 104 studies were included, covering case-control, cross-sectional, cohort, and retrospective studies from India, Europe, the Middle East, and North America. Several studies have reported higher rates of oral fungal colonization among denture wearers,with *Candida albicans* being the most frequently isolated species, followed by resistant strains such as *Candida auris*. However, these observations are primarily derived from heterogeneous observational studies and should therefore be interpreted as associative rather than causal. COVID-19-related mucormycosis (CAM) was primarily reported as rhino-orbito-cerebral disease, with oral manifestations including palatal necrosis, gingival ulcers, and tooth mobility. Key risk factors identified include diabetes mellitus, corticosteroid therapy, prolonged intensive care unit (ICU) stays, and poor denture hygiene. Mortality related to CAM ranged from 18% to 56%, while candidiasis, though less deadly, significantly affected oral function, nutrition, and overall quality of life. Diagnostic methods included clinical and intraoral examinations, microbiological cultures, imaging techniques, and emerging salivary biomarkers. Treatments included systemic antifungal medications, surgical removal, and prosthesis disinfection, highlighting the important role of prosthodontists in prevention and rehabilitation. Knowledge gaps remain regarding the predictive value of oral lesions for systemic infections, the long-term effects of COVID-19 on the oral microbiome, and the need to standardize denture hygiene protocols. This review emphasizes the importance of integrated dental and medical care in reducing morbidity and mortality among denture-wearing older adults recovering from COVID-19, while recognizing that early oral findings may serve as warning indicators rather than definitive predictors of systemic infection.

## Introduction and background

The coronavirus disease 2019 (COVID-19) pandemic has caused significant systemic and oral health effects, particularly among older adults who wear dentures. In addition to the initial SARS-CoV-2 (severe acute respiratory syndrome coronavirus 2) infection, some patients develop long COVID or post-acute sequelae of SARS-CoV-2 infection (PASC), characterized by persistent or new symptoms affecting various organ systems, including the pulmonary, cardiovascular, neurological, and immune systems [1]. Among these complications, opportunistic fungal infections, notably mucormycosis and *Candida* infections, have become severe and potentially life-threatening, with important implications for oral health and prosthodontic care [2-4].

*Candida* infections frequently co-occur with mucormycosis in immunocompromised individuals, particularly older adults with conditions such as diabetes, iron or zinc deficiency, or exposure to corticosteroids or prolonged stays in intensive care units [5-9]. Oral candidiasis typically presents as white plaques, burning, and dysphagia, while systemic spread can cause fever, chills, and sepsis [5]. Dentures increase the risk, as they can act as reservoirs for fungal biofilms, especially when oral hygiene is poor, dentures are ill-fitting, or xerostomia is present-conditions common among the elderly [10-12]. SARS-CoV-2 infection itself may disrupt oral homeostasis and cause salivary gland inflammation, thereby facilitating colonization by opportunistic fungi [10,5].

Mucormycosis, commonly known as “black fungus,” is a rapidly angioinvasive infection caused by fungi of the order Mucorales, which proliferate in environments characterized by high glucose levels, low oxygen availability, and immunosuppression [13]. The increase in COVID-19-associated mucormycosis (CAM), particularly in countries with high diabetes prevalence, such as India, has been linked to immune dysregulation, corticosteroid therapy, prolonged intensive care unit (ICU) stays, mechanical ventilation, malnutrition, and broad-spectrum antibiotic use [14-17]. Clinically, CAM predominantly presents as rhino-orbito-cerebral mucormycosis (ROCM), characterized by facial pain, sinusitis, orbital swelling, black nasal crusting, and necrosis. Oral manifestations may include palatal ulcers, gingival necrosis, and tooth mobility [18,19]. Severe cases can extend to involve the brain, leading to life-threatening complications.

Dentures may promote biofilm formation, creating persistent reservoirs for *Candida* and other fungal spores, thereby potentially worsening the severity and progression of mucormycosis in older individuals. The pathophysiology of CAM in COVID-19 patients is complex. Corticosteroid-induced immunosuppression, hyperglycaemia caused by diabetes or steroid therapy, prolonged mechanical ventilation, and increased serum iron levels all promote fungal growth and tissue invasion [9,18,20]. An imbalance in immune regulation associated with long COVID further increases the risk of opportunistic infections [1,21]. Management involves early diagnosis, prompt surgical removal of infected tissues, and antifungal treatment with agents such as liposomal amphotericin B or posaconazole, alongside careful glycaemic control and cautious use of corticosteroids [14,22]. Diagnostic imaging techniques, including contrast-enhanced magnetic resonance imaging or computed tomography (CT), help identify characteristic areas of necrotic tissue, such as the “black turbinate” sign [22].

Oral health considerations are central to the prevention and early detection of fungal complications. Dentists and oral healthcare professionals play a vital role in recognizing early signs such as gingival necrosis, palatal ulcers, and candidal plaques, which may precede serious systemic issues [6]. In older adults wearing dentures, proactive oral care, strict hygiene protocols, and routine monitoring are particularly essential because of the combined risks of prosthesis-related biofilm accumulation, immune system impairments, and systemic health conditions. Evidence indicates that early detection of oral mucormycosis or candidiasis in high-risk patients can significantly reduce morbidity and mortality, thereby emphasizing the importance of coordinated dental and medical care.

Despite growing recognition of COVID-19-associated mucormycosis and *Candida* infections, older adults who wear dentures remain under-investigated, despite heightened vulnerability related to immune senescence, systemic disease, and prosthesis-associated biofilm formation. Given the heterogeneity of available evidence and the emerging nature of this topic, a narrative review methodology was chosen to systematically map existing data, identify research gaps, and inform future investigations. Oral manifestations may signal early systemic fungal invasion; however, there is limited evidence linking denture use, long COVID, and opportunistic fungal infections. Understanding these interactions is vital for improving early detection, developing preventive strategies, enhancing clinical outcomes, and guiding dental and medical practitioners managing high-risk patients [5,11,10].

This narrative review aimed to assess the relationship between denture use, oral health, and the development of mucormycosis and *Candida* infections in older adults following COVID-19, with a particular focus on prevalence, risk factors, clinical features, diagnostic methods, and management strategies.

The study examined the prevalence and incidence of mucormycosis and oral *Candida* infections in older adults who wear dentures following COVID-19. It identified systemic and local risk factors, including diabetes, corticosteroid therapy, ICU admission, malnutrition, and denture-related issues. The study described clinical features, including palatal ulcers, gingival necrosis, and candidal plaques. It also evaluated diagnostic methods, such as histopathology, fungal cultures, and imaging techniques, for early detection. Furthermore, it examined management strategies, including surgical intervention, antifungal therapy, glycemic control, and prosthesis care. The research provided evidence-based recommendations for the early recognition, prevention, and management of fungal infections in this population, while highlighting gaps to inform future research.

## Review

Materials and methods

For this narrative review, the Patient, Intervention, Comparison, Outcome (PICO) framework was used to define the research question and guide the literature search [23]. The population of interest comprised adults (typically aged 60 years or older) diagnosed with COVID-19, particularly those who wore dentures and subsequently developed mucormycosis, oral *Candida* infections, or other oral health issues during or after the acute phase of COVID-19. Given the heterogeneity of study designs, populations, and outcomes, and the distinct biological behaviors of candidiasis and mucormycosis, a narrative synthesis was conducted using two parallel analytical tracks, with integration limited to the Discussion section.

The main exposures were SARS-CoV-2 infection, potential denture use, and related immune and oral health changes in older adults. Comparisons were made between older COVID-19 patients with fungal or oral complications and those without, and with non-COVID older adult controls, where available. The primary outcomes examined included the incidence, prevalence, risk factors, clinical features, anatomical involvement, diagnostic methods, and treatment options for mucormycosis and *Candida* infections, with particular attention to oral manifestations and denture-related factors. The PICO approach supported the investigation of the hypothesis that, in combination with older age and denture use, COVID-19 increases susceptibility to opportunistic fungal infections.

A systematic literature search was conducted across PubMed/MEDLINE, Scopus, Web of Science, Embase, and the Cochrane Library, covering publications from December 2019 to October 2025. The search strategy combined Medical Subject Headings (MeSH) terms and keywords using Boolean operators: ("COVID-19" OR "SARS-CoV-2" OR "coronavirus" OR "Long COVID" OR "post-COVID syndrome") AND ("mucormycosis" OR "zygomycosis" OR "black fungus" OR "Candida" OR "oral candidiasis") AND ("oral health" OR "oral manifestation" OR "dental" OR "periodontal" OR "stomatological" OR "maxillofacial" OR "teeth loosening" OR "dry mouth" OR "xerostomia" OR "palatal necrosis" OR "oral ulcer" OR "denture" OR "prosthesis") AND ("older adults" OR "elderly" OR "geriatrics" OR "aged 60+" OR "aged 65+"). Reference lists of included studies were also manually reviewed to identify further relevant publications.

This review included all original research studies reporting outcomes for older adults with confirmed COVID-19 infections who subsequently developed mucormycosis, oral *Candida* infections, or oral health complications, particularly among denture wearers. Eligible study designs included case series (five or more cases), cross-sectional, case-control, and cohort studies, as well as clinical trials published in English. Exclusion criteria included case reports with fewer than five patients, reviews without original data, studies that focused exclusively on extraoral fungal infections, studies with inadequate methodology or insufficient outcome reporting, and letters to the editor, commentaries, and opinion pieces without substantive data, so as to ensure methodological consistency and limit interpretive bias.

**Figure 1:**
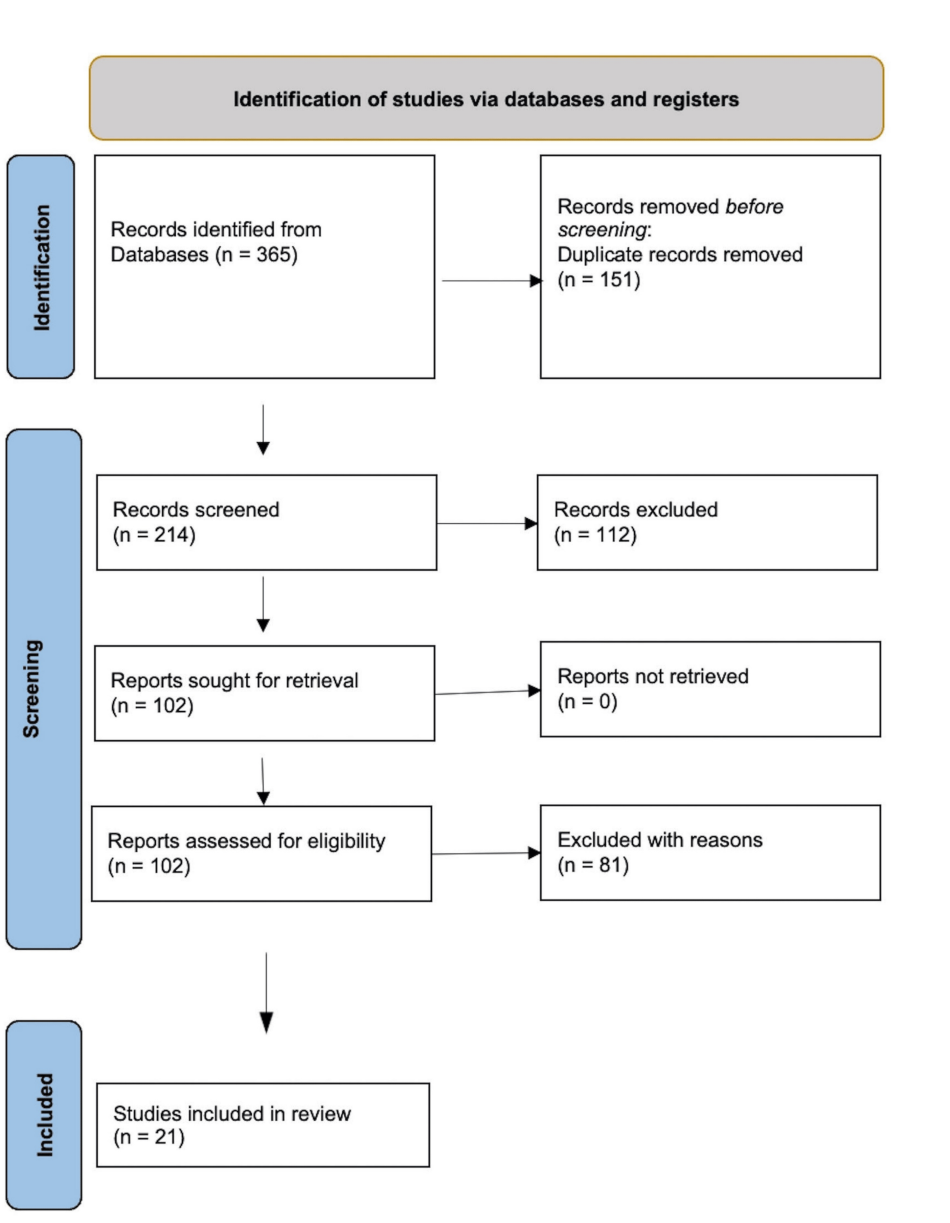
PRISMA flow chart PRISMA: Preferred Reporting Items for Systematic reviews and Meta-Analyses [25].

Two independent reviewers screened all titles and abstracts, retrieved full-text articles of potentially relevant studies, and assessed eligibility separately. Discrepancies were resolved by consensus or consultation with a third reviewer. The selection process was documented in line with the PRISMA-ScR (Preferred Reporting Items for Systematic reviews and Meta-Analyses extension for Narrative Reviews) [24,25] flow diagram guidelines (Figure 1).

Although the present work is a narrative review, the PICO and the PRISMA frameworks were used pragmatically to structure the search strategy and ensure transparent study identification, rather than to imply a formal comparative or interventional analysis.

Data extraction was performed using a standardized form that recorded: study characteristics (author, year, country, study design, sample size), patient demographics (age, gender, comorbidities, denture use), COVID-19 features (severity, treatment, duration), fungal infection details (prevalence, type-mucormycosis or *Candida*, clinical presentation, anatomical sites, diagnostic methods), oral health outcomes, risk factors (diabetes, steroid use, denture-related hygiene, xerostomia), treatment strategies (antifungal therapy, surgical intervention, prosthesis management), and statistical measures (odds ratios, confidence intervals, p-values).

Given the heterogeneity of study designs, populations, and outcomes, a narrative synthesis of the findings was conducted. Data were organized into themes, including epidemiology, risk factors, clinical manifestations, denture-specific considerations, older adult-specific factors, diagnostic methods, treatment strategies, and outcomes for mucormycosis and oral *Candida* infections in older adults.

Results

**Figure 2:**
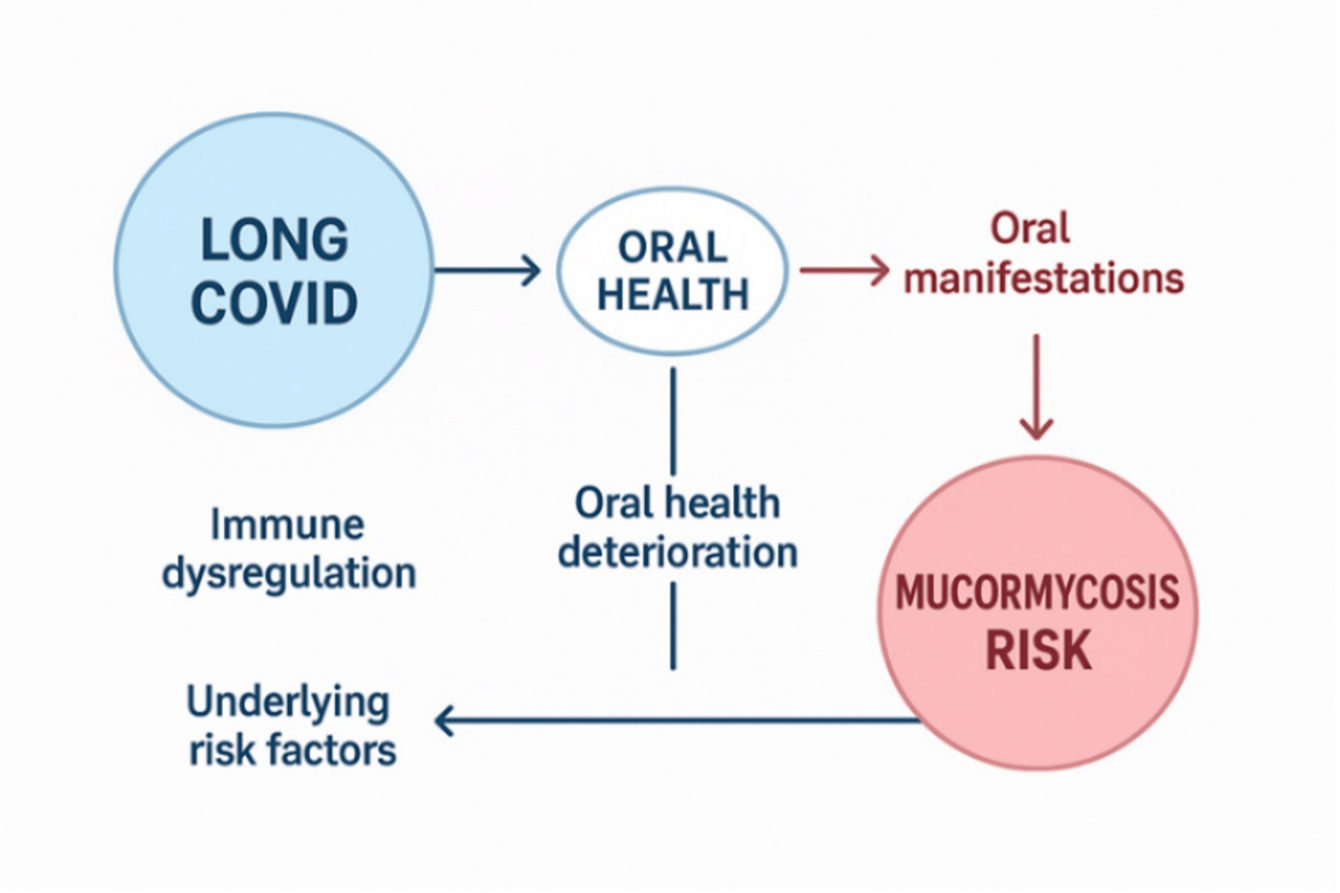
Conceptual framework linking Long COVID, oral health deterioration, and risk of mucormycosis.

Thirteen studies on candidiasis and nine on mucormycosis (with one study addressing both conditions, making it a total of 21) were included, published between 2020 and 2025. These studies employed case-control, cross-sectional, retrospective, and case-series designs and were conducted in India, Sweden, Canada, and other countries. The research focused on COVID-19-associated candidiasis and mucormycosis, as well as oral health and Long COVID, emphasizing prevalence, risk factors, clinical features, diagnostic methods, management strategies, and outcomes (Figure 2).

Candidiasis is among the most frequently reported oral symptoms in patients with COVID-19 across various regions and study designs. Alhamed et al. [26] reported candidal infections in 68% of hospitalized patients, with a significantly higher prevalence in individuals aged ≥60 years (80%, p=0.008), highlighting older adults' increased vulnerability to opportunistic fungal infections.

More specifically, Favia et al. (2021) [27] reported candidiasis in 28 of 123 patients (14.6%), mainly during the early stages of infection before COVID-19-specific treatments began, indicating that fungal colonization may occur alongside or precede severe systemic symptoms. Binmadi et al. [28] found oral manifestations in 29% of their cross-sectional cohort (with 6% candidiasis), while Gogotishvili et al. [29] detected it in 40% of patients. Sultan et al. [30] demonstrated that *Candida albicans* was present in approximately 50% of hospitalized COVID-19 patients, whereas it was absent in healthy controls, thereby reinforcing the pathogen's opportunistic nature in the context of COVID-19-induced immune dysregulation. These results collectively suggest that candidal infections are relatively common among COVID-19 patients and can occur early in the disease course. Additional studies highlighted the importance of *Candida* colonization in both hospitalized and community-dwelling COVID-19 patients. Villarroel-Dorrego et al. reported that among 55 COVID-19 patients, 22 had oral lesions; dysgeusia affected 33, burning mouth 20, xerostomia 15, while candidiasis and ulcers occurred in seven patients each. [31]. In elderly populations, Johansson et al. observed that individuals with dentures had a higher prevalence of orofacial long-COVID symptoms, suggesting persistent colonization or delayed manifestation of candidiasis in the post-acute phase [1]. Although denture hygiene practices were reported, they were not uniformly assessed or standardized, limiting interpretation of their potential confounding effect.

Clinically, candidiasis in COVID-19 patients presented with a range of signs and symptoms. Alhamed et al. documented white patches and intraoral ulcerations on the tongue, buccal mucosa, and palate [26]. Favia et al. (2021) [27] identified candidiasis among various oral lesions, including ulcerative lesions, blisters, papillary hyperplasia, and angina bullosa, highlighting the diverse oral manifestations associated with COVID-19.

Additional clinical features included erythema, depapillation, atrophic glossitis, enanthems [29] and angular cheilitis [32]. Mahmoud et al. reported glossitis, erythematous erosions, and aphthous ulcerations [33]. These presentations were often associated with taste alterations and xerostomia, indicating that candidal infections in COVID-19 patients are frequently coupled with other oral sensory disturbances [26,29]. Collectively, these findings demonstrate that candidiasis may present as isolated white patches or ulcers, or coexist with multifocal oral lesions, reflecting the complex oral pathophysiology induced by SARS-CoV-2 infection.

Several studies have identified patient- and disease-specific risk factors for candidal infections in COVID-19. Advanced age consistently emerged as an associated predictor, with Alhamed et al. reporting an 80% prevalence of candidiasis in patients aged ≥60 years [26]. Immunocompromised status and systemic comorbidities, including cardiovascular disease and diabetes, were implicated by Jerônimo et al. and Villarroel-Dorrego et al., who noted that patients with prosthetic stomatitis and poor oral hygiene were particularly vulnerable [8,31].

Favia et al. (2023) identified oral lesions as predictors of severe disease, with a relative risk (RR) of 7.998 (p=0.002), while autoimmune disease was associated with an odds ratio (OR) of 8.838 for adverse outcomes [34]. In long-COVID survivors, Johansson et al. (2024) highlighted denture use as a strong predictor of orofacial symptoms, with ORs of 3.4-5.0, emphasizing the association between prosthetic devices, oral microbial colonization, and COVID-19 complications [1]. In aggregate, these findings indicate that older age, systemic comorbidities, immunosuppression, ICU admission, and denture use increase susceptibility to candidiasis, with immune dysregulation caused by SARS-CoV-2 serving as a common underlying mechanism.

Treatment approaches for COVID-19-associated candidiasis have been reported inconsistently. In most observational and cross-sectional studies, candidiasis was diagnosed by intraoral examination and microbiological sampling, but therapeutic interventions were often not systematically recorded [26-28]. Villarroel-Dorrego et al. confirmed that most *Candida* isolates were susceptible to azoles, amphotericin B, and echinocandins, indicating that standard antifungal regimens are generally effective [31].

Santos et al. recommended rigorous oral care protocols for denture wearers to prevent infection and reduce morbidity, emphasizing prophylaxis as a critical adjunct to pharmacological treatment [35]. Overall, these findings suggested that a dual approach to candidiasis management in COVID-19 is needed: timely pharmacological intervention and diligent oral care, particularly in high-risk populations. COVID-19 amplifies *C. albicans* colonization in complete denture wearers, with higher counts in severe cases; the duration of denture use strongly correlates with infection risk [35]. 

Studies reporting oral manifestations of COVID-19 are summarized in Table 1.

**Table 1:** Summary of studies reporting oral manifestations in COVID-19 patients. The table organizes studies by author, year, country, study design, sample characteristics, COVID-19 severity, observed symptoms, treatments, outcomes, and key risk factors. It highlights the prevalence of oral lesions, including candidiasis, ulcers, taste disorders, and xerostomia, and emphasizes associations with disease severity, age, comorbidities, and denture use.

Author(s)	Year	Country	Study Design	Sample Size	Age (Years)	COVID-19 Severity	Symptoms (%)	Treatment (%)	Outcomes (%)	Key Risk Factors (OR or %)
Johansson et al. [1]	2024	Sweden	Questionnaire	5375 (follow-up 577)	80-90	Acute and Long COVID	*Acute*: general: 88%, orofacial: 44%; *Long*: general: 37%, orofacial: 35%; Altered taste, 60%; burning mouth, 36.4%; dry mouth, 27.3%	Questionnaire	Long-COVID oral/general symptoms associated with dentures	Risk factors: elderly housing (OR 1.6); >10 social contacts/week (OR 1.5); married (OR 1.4); higher education (OR 1.3). Long-COVID: denture use (OR 5.0), bad breath (OR 3.7–3.8), dry mouth (OR 2.2).
Alhamed et al. [26]	2023	Saudi Arabia	Case-control	22 (M=16, F=6)	57.9±16.1	Moderate to Severe	Candidal infections 68%, oral ulcerations 36%, white patches 27.3%, taste/smell loss 65%, oral dryness 45%, halitosis 30%	Not reported	Candidal infection and taste disturbance are the most frequent	Candidal infections in ≥60 years: 80% (p=0.008)
Favia et al. [27]	2021	Italy	Observational	123 (M=70, F=53)	Median 72	Moderate 77%, Severe 17%, Critical 6%	Ulcerative lesions 65, blisters 19, candidiasis 28, geographic tongue 7	Intraoral exam, cytology	Early lesions in 65.9% before therapy may indicate peripheral thrombosis	Histopathology: thrombosis in early lesions
Binmadi et al. [28]	2022	Saudi Arabia	Cross-sectional	195 (M=48, F=147)	18-55+	Mild 21%, Moderate 59%, Critical 20%	Oral manifestations 29%,oral ulcers 11%, dysgeusia 60%, 6% candidiasis xerostomia 42%,	Patient interview & history	Oral changes are common,especially in women	25% smokers, 33% takenmedications,24% hospitalized
Gogotishvili et al. [29]	2024	Georgia	Cohort	55 (M=30, F=25)	18-89	Mild-Moderate	Candidiasis & oral lesions ulcers 40%, angular cheilitis, enanthems, geographic/caviar tongue	Intra/extra oral exam	Oral mucosal alterations are prevalent	Complete dentures (general), (orofacial); bad breath dry mouth
Villarroel-Dorrego et al. [31]	2020	Venezuela	Observational clinical descriptive (case series)	55 (M=30, F=25)	1-89	55 Hospitalized	Altered taste, dry mouth, and painful/burning mouth were noted in 60%, 27.3%, and 36.4% of patients, respectively	Species ID & antifungal susceptibility	*Candida albicans,* ulcers	All hospitalized ICU patients (34,5%) received dexamethasone/remdesivir; ward patients mostly received lopinavir/ritonavir therapy.
Ganesan et al. [32]	2022	India	Cross-sectional	500 (M=367, F=133)	53.46± 7.50	Mild	Gustatory disturbances 51.2%, xerostomia 28%, oral lesions 15.4%	Intra/extra oral exam	Oral manifestations correlated with disease severity	Statistically significant presence of oral manifestations with severe disease (p≤0.001)
Mahmoud et al. [33]	2022	Egypt	Case-series	5 (M=2, F=3)	3-32	Not reported	Taste alteration, petechiae, gingivitis,, vague ulcerations. glossitis, candidiasis,	Clinical & intra/extra oral exam	Emphasised the mandatory oral exam in COVID-19	Impaired immune system, coinfections, side effects related to treatments
Favia et al. [34]	2023	Italy	Cross-sectional	103 (M=46,6%, F=53,4%)	69.94±10.99	Mild-Moderate	Single/multiple ulcers, petechiae, candidiasis	Intra/extra oral exam	Higher risk of severe disease with oral lesions	Oral lesions related to Covid-19 RR=7.998, p=0.002; Autoimmune disease OR=8.838, p=0.026
Ali et al. [36]	2023	India	Observational study	45 complete denture wearers (15 mild-moderate COVID-19, 15 severe COVID-19, 15 controls)	50-60 years old	Mild-moderate, Severe, and Non-COVID groups	Not specified	Not specified	Higher *Candida albicans* CFU in severe COVID-19 > mild-moderate COVID-19 > controls; significant (p<0.001)	Strong positive correlation between *C. albicans* count and duration of denture use; weak positive correlation with age; COVID-19 compounded association
Babamahmoodi et al. [37]	2023	Iran	Observational study	4,133 COVID-19 patients admitted; 120 (2.9%) developed oral candidiasis	Not specified	Moderate to severe hospitalized COVID-19	100% of Oral candidiasis patients (OC patients) (2.9% of total sample); most common=white/yellow macules on buccal surface or tongue	Corticosteroids, broad-spectrum antibiotics, nasal corticosteroid spray (usage percentages not specified)	Majority recovered; better recovery in single-species OC vs multi-species (P=0.049)	Multiple *Candida *strains are more common with corticosteroids (P<0.0001), antibiotics (P=0.028), and nasal corticosteroid spray (P<0.0001), oral candidiasis caused by multiple *Candida* strains
Ohara et al. [38]	2023	Japan	In vitro experimental study	Not specified	Not applicable	Not applicable	Not applicable	Hinora® (hinokitiol+IPMP) vs Refrecare® (hinokitiol only)	Oral care gel -containing and hinokitiol inhibited biofilm formation and growth of *Candida* spp. and multiple bacteria at ≥0.05 g/mL; suppressed *C. albicans* germ tube formation	Elderly with dentures, reduced saliva, weakened immunity, and *Candida* overgrowth face higher oral infection and aspiration pneumonia risks
Buendia et al. [39]	2024	USA	Cross-sectional observational study (CLAIRE study)	120 hospitalized patients (116 COVID-19 positive)	Not specified	Mild, severe, critical (classified retrospectively)	Not specified	Broad-spectrum antibiotics (BSA) given to all; higher use in suspected sepsis cases	12 deaths, all in BSA group; salivary microbiome altered by BSA	BSA use linked to mortality; *C. albicans* detected most in critical patients; Staphylococcus aureus risk factor for sepsis (non-BSA group)

Outcomes of candidiasis in COVID-19 patients varied according to patient risk profiles and disease severity. In hospital-admitted cohorts, candidal infections were frequently associated with taste disturbances and ulcerations [26], and in some cases indicated increased susceptibility to severe COVID-19 [34]. ICU patients and those with comorbidities, such as autoimmune disease, exhibited higher morbidity and were at risk of complications due to immune dysregulation [8,31].

Gogotishvili et al. reported that candidiasis frequently co-occurs with painful or burning mouth sensations, xerostomia, and altered taste, which contribute to reduced oral comfort and may affect nutrition [29]. Long-term follow-up studies, such as those by Johansson et al., have shown that denture-associated oral changes can persist in post-acute COVID-19 patients, suggesting that candidiasis may contribute to chronic or recurrent orofacial symptoms [1]. Overall, the findings emphasize candidiasis as both a direct oral health concern and a potential marker for severe or prolonged COVID-19 complications, underscoring the importance of proactive monitoring and management in at-risk populations.

Additional studies are summarized in Tables 2, 3 and support this review [36-42]. Opportunistic fungal infections, predominantly *C. albicans*, with bacterial or viral co-infections were common [36,37]. Lesions primarily affected the tongue, palate, and buccal mucosa, presenting as ulcers and angular cheilitis [39]. Knowledge gaps persist due to limited longitudinal data. Polymicrobial interactions influenced severity [40-43]. ICU patients experienced higher morbidity, while diagnostics focused on saliva-based and molecular methods [41,42], and management emphasized antifungals and oral care [42-48].

**Table 2:** Summary of COVID-19-associated oral candidiasis: key findings on risk factors, oral manifestations, co-infections, diagnostics, treatment, and knowledge gaps, compared with published studies.

Theme	Findings from Results	Comparison with Literature
Risk factors	Age ≥60, ICU admission, autoimmune disease, comorbidities (diabetes, cardiovascular), denture use (Johansson et al., 2024 [1]; Alhamed et al., 2023 [26]; Villarroel-Dorrego et al., 2020 [31])	Older age, comorbidities, ICU stay, corticosteroid use, and denture wearing consistently identified as major risk factors in multiple studies (Ohashi et al., 2021 [7]; Jerônimo et al., 2021 [8]; Alfaifi et al., 2024 [16]; Ali et al., 2023 [36]; Babamahmoodi et al., 2023 [37])
Anatomical distribution	Tongue, palate, buccal mucosa; angular cheilitis, enanthems, geographic tongue (Gogotishvili et al., 2024 [29]; Huang et al. [40])	Observational studies and reviews report tongue, palate, and buccal mucosa as primary sites; angular cheilitis and oral ulcers common in severe or ICU patients (Ohashi et al., 2021 [7]; Favia et al., 2021 [27]; Alfaifi et al., 2024 [16]; Cuevas-Gonzalez et al. [41]; Favia et al., 2023 [34])
Oral manifestations	Candidal infections (18–68%), oral ulcers, white patches, taste/smell disturbances, xerostomia, burning mouth (Alhamed et al., 2023 [26]; Binmadi et al., 2022 [28]; Gogotishvili et al., 2024 [29]; Ganesan et al., 2022 [32])	Literature confirms these manifestations are frequent, especially in hospitalized and older adults (Ohashi et al., 2021 [7]; Favia et al., 2021 [27]; Alfaifi et al., 2024 [16]; Cuevas-Gonzalez et al. [41]; Favia et al., 2023 [34]; Ali et al., 2023 [36]; Ohara et al., 2023 [38])
Candidiasis co-infection	*Candida albicans* most frequent; non-*albicans* *Candida* reported; co-infection with herpes simplex, bacterial pathogens (Sultan et al., 2021 [30]; Santos et al., 2020 [35]; Babamahmoodi et al., 2023 [37])	Opportunistic fungal infections frequently reported due to immune dysregulation, corticosteroids, or broad-spectrum antibiotics (Nambiar et al., 2021 [9]; Alfaifi et al., 2024 [16]; Alfaifi et al., 2022 [42]; Gregorczyk-Maga et al. [43]; Küçükkaya et al. [44])
Mortality and morbidity	Associated with pain, dysphagia, reduced oral intake, impaired Quality of Life; mortality impact unclear (Alhamed et al., 2023 [26]; Santos et al., 2020 [35])	ICU and mechanically ventilated patients have increased morbidity; candidiasis can worsen systemic infection but rarely causes death directly (Katz, 2021 [6]; Jerônimo et al., 2021 [8]; Alfaifi et al., 2024 [16])
Diagnostic approaches	Clinical intraoral exam, scraping/culture, microbiome profiling, questionnaires (Johansson et al., 2024 [1]; Binmadi et al., 2022 [28]; Villarroel-Dorrego et al., 2020 [31])	Combination of clinical and microbiological methods widely used; saliva-based diagnostics and molecular profiling emerging (Sultan et al., 2021 [30]; Alfaifi et al., 2024 [16]; Ohara et al., 2023 [38]; Buendia et al., 2024 [39]; Lam et al., 2025 [45]; Ramezanalipour et al. [46])
Treatment Strategies	Symptomatic management, antifungals, pain control, oral care gels; emphasis on ICU dental support (Santos et al., 2020 [35]; Ohara et al., 2023 [38]; Al-Kenani et al. [47])	Topical/systemic antifungals, maintenance of oral hygiene, prophylaxis in high-risk patients; novel delivery systems explored (Ohashi et al., 2021 [7]; Alfaifi et al., 2024 [16]; Peng et al., 2024 [48]; Lam et al., 2025 [45])
Mixed infections and ecology	Altered oral microbiome, reduced bacterial diversity, increased fungal colonization; antibiotics shift oral ecology (Sultan et al., 2021 [30]; Alfaifi et al., 2024 [16]; Buendia et al., 2024 [39])	Dysbiosis favoring *Candida* overgrowth observed in hospitalized patients; polymicrobial interactions influence disease severity (Nambiar et al., 2021 [9]; Alfaifi et al., 2024 [16]; Alfaifi et al., 2022 [42]; Gregorczyk-Maga et al. [43])
Geographical and temporal variability	Data from Saudi Arabia, Italy, India, Egypt, USA, Sweden, Iran; manifestations across mild–severe COVID-19 and long-COVID (Johansson et al., 2024 [1]; Alhamed et al., 2023 [26]; Ganesan et al., 2022 [32])	Global prevalence documented; variations depend on healthcare access, ICU use, corticosteroids, and age (Katz et al., 2021 [6]; Jerônimo et al., 2021 [8]; Alfaifi et al., 2024 [16]; Ali et al., 2023 [36]; Cuevas-Gonzalez et al. [41])
Knowledge gaps	Limited longitudinal data; inconsistent reporting of severity, treatments, and microbiome shifts; need standardized diagnostic criteria	Literature highlights lack of large-scale longitudinal studies, unclear mechanisms, and limited evidence on effective prophylactic/therapeutic interventions (Alfaifi et al., 2024 [16]; Cuevas-Gonzalez et al. [41]; Alfaifi et al., 2022 [42])

**Table 3:** Summary of studies reporting oral and maxillofacial manifestations, treatments, and outcomes in COVID-19 and COVID-19-associated mucormycosis (CAM) patients. PCR: Polymerase chain reaction; OR: odds ratio; ROCM: rhino-orbito-cerebral mucormycosis; AAI: Appearance Anxiety Inventory; PHQ9: Patient Health Questionnaire-9; FESS: functional endoscopic sinus surgery.

Author(s)	Year	Country	Study Design	Sample Size	Age (Years)	COVID-19 Severity	Symptoms (%)	Treatment (%)	Outcomes (%)	Key Risk Factors (OR or %)
Johansson et al. [1]	2024	Sweden	Questionnaire / survey (cross-sectional for symptoms + retrospective predictive analysis using pre-COVID data)	• 5,375 total (577 reported COVID-19). • COVID questionnaire: ~278.	80- and 90-year-olds	Mixed (self-reported mild/moderate vs severe/very severe; some hospitalized)	Acute: ~88% general; ~44% orofacial. Long-COVID: ~37% general; ~35% orofacial.	Treatment not specified (focus: symptoms & risk factors).	Outcomes: prevalence of acute and long-COVID general/orofacial symptoms. No mortality outcomes reported.	Risk factors: elderly housing (OR 1.6); >10 social contacts/week (OR 1.5); married (OR 1.4); higher education (OR 1.3). Long-COVID: denture use (OR 5.0), bad breath (OR 3.7–3.8), dry mouth (OR 2.2).
Alfaifi et al. [16]	2024	USA & Saudi Arabia	Clinical + microbiome analysis (cross-sectional case–control oral microbiota study)	• 47 participants: 26 COVID-19 positive, 21 controls	Adults (general population)	PCR-confirmed COVID-19; severity not stratified	Not symptom focused. Microbiota findings: ↑ prevalence of *Candida albicans* and oral dysbiosis in COVID-19 subjects.	No treatment reported (microbiome profiling only).	Outcomes: significant oral microbiota dysbiosis, opportunistic fungal overgrowth in COVID-19 patients.	Risk factor: COVID-19 infection strongly associated with oral dysbiosis;* Candida albicans* identified as potential opportunistic risk for oral/systemic complications.
Somkuwar et al. [17]	2023	India	Cross-sectional observational study at a tertiary care hospital	• 177 patients with COVID-19–associated mucormycosis (CAM)	Wide adult age range (mean ~50s; both sexes)	Confirmed COVID-19 patients with secondary mucormycosis (majority moderate–severe; many with hospitalization & steroid use)	Oral manifestations: ~43% had oral findings. Common: palatal discoloration/necrosis, loosening of teeth, oro-nasal fistula. Other systemic symptoms: orbital/facial pain, nasal congestion, headache, swelling.	Standard treatment: antifungal therapy combined with surgical debridement where necessary.	Outcomes: significant morbidity; high rates of surgical intervention; survival dependent on early diagnosis and combined antifungal + surgical management.	Risk factors: diabetes mellitus (majority of cases), prolonged corticosteroid therapy, uncontrolled hyperglycemia, and prior hospitalization for severe COVID-19.
Bansal et al. [12]	2022	India	Retrospective observational study (COVID-associated mucormycosis in kidney transplant recipients)	• 11 kidney transplant recipients with CAM	Adults (mean ~50s; transplant population)	All confirmed COVID-19 cases post-kidney transplantation (moderate–severe; high immunosuppression burden)	Oral/craniofacial & systemic features: rhino-orbital mucormycosis predominant; headache, facial pain/swelling, orbital involvement, sinus disease. Oral mucosal/ palatal necrosis in some cases.	Treatment: antifungal therapy (liposomal Amphotericin B), surgical debridement, reduction of immunosuppression.	Outcomes: very high mortality (~45–50%); survivors required aggressive combined antifungal + surgical treatment.	Risk factors: kidney transplantation, immunosuppressive therapy, diabetes, corticosteroid exposure during COVID-19, and severe COVID infection itself.
Desai et al. [49]	2022	India	Retrospective observational study of post-COVID rhino-orbital mucormycosis	• 100 patients with post-COVID rhino-orbital mucormycosis	Adults (mean ~51-60 years)	All post-COVID; severity: moderate–severe COVID-19 requiring hospitalization	Oral/craniofacial & systemic features: orbital/facial pain and swelling, nasal congestion, proptosis, palatal involvement/necrosis.	Standard treatment: antifungal therapy (Amphotericin B), surgical debridement as indicated.	Outcomes: most patients recovered with combined medical + surgical management; some morbidity due to orbital involvement; no mortality reported.	Risk factors: uncontrolled diabetes mellitus, corticosteroid therapy during COVID-19, delayed presentation, prior hospitalization for severe COVID-19.
Moorthy et al. [50]	2021	India	Retrospective, multi-centric observational study of invasive maxillofacial fungal infections in post-COVID patients	• 18 patients with invasive fungal infections (mostly rhino-orbito-cerebral mucormycosis)	Adults (age range 35–70)	All post-COVID: most moderate–severe, many treated with corticosteroids	Oral/craniofacial & systemic features: facial/orbital pain and swelling, palatal necrosis, sinus involvement, orbital proptosis, nasal congestion, headache	Standard treatment: antifungal therapy (Amphotericin B), surgical debridement, supportive care	Outcomes: high morbidity; combined antifungal + surgical management improved survival; mortality reported ~15–20%	Risk factors: uncontrolled diabetes mellitus, corticosteroid therapy, delayed diagnosis, COVID-19 infection severity, hypoxia
Joshi et al. [51]	2021	India	Retrospective single-center imaging analysis (CT and MRI)	Imaging review of 25 patients with COVID-associated invasive mucormycosis	Adults (34-76 years)	All post-COVID; mostly moderate–severe COVID-19 requiring hospitalization	Radiological/orofacial features: sinus opacification, orbital invasion, bone erosion, palatal involvement; clinical symptoms included facial pain, swelling, orbital symptoms	Not treatment-focused (imaging study only)	Outcomes: early imaging facilitated diagnosis; improved surgical planning and management; no direct mortality data reported	Risk factors: diabetes mellitus, corticosteroid therapy, severe COVID-19, hypoxia, delayed diagnosis
Naddaffard et al. 2025 [52]	2025	Iran	Single-center observational study	57 mucormycosis patients with confirmed cultures	Mean age 56.03 years	COVID-19-associated cases (severity not detailed)	Periorbital edema (48%), decreased visual acuity (28%)	Amphotericin B (93%), posaconazole (75%), surgical intervention (86%, mostly Functional Endoscopic Sinus Surgery FESS 61%)	26% mortality	Diabetes mellitus (74%), corticosteroid use, immunosuppression, COVID-19 infection
Sindi et al. 2024 [53]	2024	India	Prospective cohort study	60 post-mucormycosis maxillectomy patients (33 males, 27 females)	Not specified	Post-COVID-19 (ROCM patients)	Appearance anxiety inventory (AAI mean 29.13±4.72 at T0); depression PHQ9 mean 16.81±4.89 at T0	Psychotherapy, pharmacotherapy, maxillofacial prosthodontic rehabilitation (obturator, implant, orbital/ocular prosthesis)	Anxiety and depression significantly reduced to normal levels at 1 year (T3: AAI 16.88±3.02; PHQ9 7.38±3.37)	Post-COVID-19 ROCM, diabetes mellitus, extent of maxillectomy, psychological distress

Somkuwar et al. (2023) [17] conducted a cross-sectional study of 171 post-COVID-19 mucormycosis patients, with a mean age of 49 years (standard deviation (SD) 10), and 71% were men. Diabetes mellitus type II was present in 47%, with a median haemoglobin A1c (HbA1c) of 9.1% (interquartile range (IQR) 7-11.1%). Only 28% had received one dose of the COVID-19 vaccine, and 2.9% were fully vaccinated. Hospitalization was required in 76% of cases, with an average stay of 11 days (SD 6.4). Treatments included steroids in 80%, antibiotics in 87%, antivirals in 51%, and oxygen therapy in 71% (39.1% received oxygen for more than seven days). Mucormycosis symptoms appeared within seven days in 16%, between eight and 30 days in 75%, and after 30 days in 9%. Oral findings included palatal necrosis, dark eschars, abscesses (38%), and perforations (20%). Desai et al. [49] studied 100 post-COVID-19 mucormycosis patients over two months. Headache and facial pain were present in 55%; hard palate involvement occurred in 45%, and unilateral disease was observed in 68%. Only 25% of early presenters had normal vision, while 22 patients experienced complete vision loss. Eye movement restriction was noted in 58%. Diabetes mellitus was the most common risk factor, accounting for 65%. Nine patients required orbital exenteration; 18% received Amphotericin for more than 14 days. Early diagnosis enhanced the prognosis.

Bansal et al. (2022) [12] reported mucormycosis in 11 out of 102 kidney transplant recipients (10.7%) following COVID-19. Six patients experienced mild COVID-19, and five experienced moderate COVID-19. Seven had pre-existing diabetes, and four developed new-onset hyperglycaemia after steroid therapy; all had poorly controlled blood sugar levels. Rhino-orbital-cerebral mucormycosis occurred in 10 out of 11 (89%), with one case of pulmonary mucormycosis.

Moorthy et al. reported 18 COVID-19 patients with maxillofacial/rhino-cerebro-orbital fungal infections. Sixteen had diabetes mellitus, 16 received systemic corticosteroids, and 15 had both diabetes mellitus and corticosteroid exposure. Mucormycosis was identified in 16 cases, aspergillosis in one case, and a mixed fungal infection in one case. Visual loss occurred in 12 patients, and seven required orbital exenteration. Outcomes included 11 survivors, six deaths, and one lost to follow-up. The incidence of diabetes was significant (p=0.03), and steroid use was significantly more frequent (p=0.0013) [50].

Alfaifi et al. reported notable alterations in the oral microbiota of hospitalized COVID-19 patients [16]. Both bacterial and viral diversity were reduced; twelve bacterial species showed a negative correlation with infection. *C. albicans* was detected in approximately 50% of patients, whereas it was absent in controls, suggesting that SARS-CoV-2-induced immune dysregulation promotes fungal overgrowth.

Joshi et al. reported imaging findings in 25 COVID-19 patients with ROCM: sinus wall erosions (n=20), intraosseous air (n=11), and focal non-enhancement of the mucosa (n = 8), along with orbital, vascular, and intracranial complications. Radiological awareness was emphasized for early detection and management [51].

Johansson et al. identified Long COVID symptoms in 37% of elderly participants (general) and 35% of elderly participants (orofacial). Acute COVID-19 symptoms were reported in 88% (general) and 44% (orofacial) [1].

*Host Factors*: Diabetes was consistently linked to mucormycosis, with Desai et al. reporting 80% [49], Moorthy et al. 89% (p=0.0013) [50], Joshi et al. 88% [51], and Bansal et al. noting that HbA1c >8.25 was significant (p<0.001) [12]. Other comorbidities included hypertension at 33%, ischaemic heart disease at 9% [48], coronary artery disease at 55% (p=0.016) [12], and HIV infection at 8% [51]. Oral health indicators, such as poor oral hygiene (OR=2.55) and grade III tooth mobility (OR = 15.8), were predictors of mucormycosis [17].

*Iatrogenic Factors*: Steroids were used in 80% [17], 89% [50], and 100% [12]; oxygen therapy was used in 71% [17]. Antibiotics (87%) and antivirals (51%) were potentially disruptive to mucosal defences.

*Social/Demographic Factors*: Elderly housing (OR=1.6), >10 weekly contacts (OR=1.5), marriage (OR=1.4), and higher education (OR=1.3) predicted Long COVID [1]. Oral health: complete dentures (OR=5.0), halitosis (OR=3.7-3.8), and dry mouth (OR=2.2).

Treatment was multimodal. Amphotericin B was administered in 82%-100% of cases. Surgical interventions included functional endoscopic sinus surgery in 61%, maxillectomy in 39%, and orbital exenteration in 39% [50]. Kidney transplant recipients (KTRs) received prophylaxis with liposomal amphotericin B and posaconazole [12]. Early diagnosis and intervention improved outcomes [52-57].

Mortality rates varied: 56% [57], 20% [53], and 18% [12]. Recovery ranged from 61% to 82%. One KTR developed acute T-cell-mediated rejection [12]. The mean serum creatinine increased from 1.4 mg/dl to 2.05 mg/dl. Long COVID persisted in 26.2%, with symptoms including fatigue, xerostomia, cough, and fever. COVID-19-associated mucormycosis results from host factors (particularly diabetes), iatrogenic interventions (steroids, oxygen, antibiotics), and oral health issues. Clinical features range from sinonasal to orbital involvement, with rapid progression and high mortality if untreated. Combining antifungal therapy with surgical debridement remains crucial. Long-term oral and systemic consequences highlight the importance of early detection, interdisciplinary care, and monitoring of post-COVID-19 patients.

Discussion

This comprehensive review highlights the increased vulnerability of older adults to COVID-19-associated opportunistic fungal infections, particularly oral candidiasis and mucormycosis, and underscores the crucial role of prosthodontic interventions in both prevention and rehabilitation. Age-related Immunosenescence, systemic conditions such as diabetes mellitus and cardiovascular disease, and the extensive use of removable dental prostheses all contribute to creating a highly susceptible oral environment [1,9,11,16]. SARS-CoV-2 infection worsens these risks by causing lymphopenia, dysregulated cytokine release, and reduced levels of antimicrobial peptides, which together promote *Candida* colonization and, in severe cases, mucormycotic invasion [5]. It should be noted that, while some of the proposed mechanisms are supported by direct clinical and experimental evidence, others are inferred from related pathophysiological and microbiological studies and therefore represent plausible biological associations rather than confirmed causal mechanisms.

Additional studies are summarized in Table 4 to further support the present review. The synthesized evidence identifies diabetes mellitus, corticosteroid exposure, and immunosuppression as the dominant risk constellation for COVID-19-associated mucormycosis (CAM), frequently termed the “unholy trinity,” with additional contributory roles for hyperglycaemia, COVID-19-directed steroid therapy, and denture use [52-57]. Although CAM predominantly affects metabolically compromised hosts, it has also been reported in non-diabetic and immunocompetent individuals, underscoring broader susceptibility [58]. Rhino-orbital-cerebral involvement remains the most common anatomical presentation, while pulmonary and mucocutaneous forms are less frequent and may occur alongside dual fungal infections, including aspergillosis [59-64]. Oral manifestations - such as necrotic lesions, periodontal abscesses, and maxillofacial involvement - often represent early diagnostic indicators, reinforcing the importance of dental surveillance [65-68]. Recurrent co-infection with candidiasis reflects polymicrobial dysbiosis, complicating diagnosis and worsening outcomes [69-73]. Mortality and morbidity remain substantial, particularly with delayed diagnosis and uncontrolled diabetes, often necessitating extensive surgical intervention [74-77]. Early diagnosis relies on clinical suspicion, imaging, and molecular tools [45,60,77-79]. Multimodal therapy combining antifungal agents with aggressive surgical debridement remains the standard, with adjunctive strategies under investigation [80]. The pronounced clustering in India highlights CAM as an “epidemic within a pandemic,” emphasizing the need for standardized reporting and the integration of oral health metrics into risk-stratification frameworks [65,77,81].

**Table 4:** Review comparison with published literature on COVID-19-associated mucormycosis and oral health.

Theme	Findings from Results	Comparison with Literature
Risk factors	Diabetes mellitus, corticosteroid use, and immunosuppression identified as major contributors to CAM, dentures.	Confirmed as the “unholy trinity” predisposing to CAM (Naddaffard et al. [52]; Safiia et al. [54]). Strongly linked to COVID-19 therapies, hyperglycemia, and steroid use (Choi et al. [55]; Sujiv et al. [56]; Ponnaiah et al. [57]). Also reported in non-diabetic and immunocompetent patients (Soleimani et al. [58]), dentures (Johansson et al. [1]).
Anatomical distribution	Predominantly rhino-orbital-cerebral involvement; pulmonary mucormycosis is less frequent.	Consistent with prior reviews (Liu et al. [59]; Rhee et al. [60]; Anitha et al. [61]). Mucocutaneous involvement reported in select cases (van der Westhuizen et al. [62]). Pulmonary involvement less common, but dual infections with aspergillosis noted (Tashiro et al. [63]; Yang et al. [64]).
Oral manifestations	Oral lesions, periodontal abscesses, and maxillofacial involvement often serve as early diagnostic indicators.	Supported by Chouksey et al. [65]; Lam et al. [45]. Periodontal and mixed infections frequently reported (Bharti et al. [66]; Pandey et al. [67]; Sharma et al. [68]). Reinforces importance of dental assessments for early CAM detection.
Candidiasis co-infection	Co-occurrence of candidiasis with mucormycosis documented in multiple cohorts.	Matches observations of mixed fungal infections (Silva et al. [69]; Zia et al. [70]; Guimarães et al. [71]). Candida predominance in oral microbiota of COVID-19 patients noted (Nguyen and Ng [72]; Alfaifi et al. [16]). Co-infections increase diagnostic complexity and impact outcomes (Brown et al. [73]).
Mortality and morbidity	High mortality and morbidity; extensive surgery associated with significant functional impact.	Agrees with Eshraghi et al. [74]; Hameed et al. [75]; Mohanty et al. [76]. Mortality worsened by uncontrolled diabetes and delayed intervention (Ahmadkhani et al. [77]). Syndemic burden highlighted in multicenter studies (Ahmadkhani et al. [77]; Soleimani et al. [58]).
Diagnostic approaches	Imaging, clinical suspicion, and early recognition critical.	Supported by Rhee et al. [60]; Hallur et al. [78]; Chouksey et al. [65]. Radiological assessment emphasized craniofacial and orbital involvement (Lam et al. [45]). Molecular diagnostics including PCR increasingly used (Brown et al. [73]; Soltani et al. [79]).
Treatment strategies	Multimodal therapy—antifungals combined with surgical debridement—improves outcomes; adjunctive therapies explored.	Consistent with Mohanty et al. [76]; Eshraghi et al. [74]; Chouksey et al. [65]. Early initiation critical (Ahmadkhani et al. [77]). Experimental adjuncts such as posaconazole, hyperbaric oxygen, and novel antifungal agents reported (Villarroel-Dorrego et al. [31]; Mohanty et al. [76]; Tanwar et al. [80]).
Mixed infections and ecology	Complex polymicrobial interactions, including Candida and Aspergillus co-infections, observed in oral and systemic sites.	Supported by Silva et al. [69]; Guimarães et al. [71]; Zia et al. [70]. Dysbiosis and immune suppression drive fungal overgrowth; oral mucosal surveillance highlighted (Nguyen and Ng [72]; Guimarães et al. [71]).
Geographical and temporal variability	Higher incidence in India and neighboring regions; temporal surges linked to COVID-19 waves and steroid use.	CAM described as an “epidemic within a pandemic” (Yadav et al. [81]; Soleimani et al. [58]). Delayed onset and regional clustering reported (Hameed et al. [75]; Bharti et al. [66]). Geographical variation tied to host susceptibility and healthcare factors.
Knowledge gaps	Limited data on oral health as predictive marker, early screening protocols, and long-term morbidity mitigation.	Echoed by Chouksey et al. [65]; Bharti et al. [66]; Tanwar et al. [80]. Need for standardized reporting, prospective studies, and integration of multidisciplinary assessments into CAM risk stratification (Ahmadkhani et al. [77]).

Elderly individuals who wear dentures form a subgroup at increased risk due to biofilm build-up on prosthetic surfaces, which act as persistent reservoirs for *Candida* species (*Candida* spp.) and promote secondary mucormycotic colonization [1,8]. In fact, colonization rates can reach up to 80% among denture wearers, underscoring the need for strict hygiene protocols and regular prosthetic evaluations [8,26]. Anatomical distribution further supports this link: candidiasis primarily affects the denture-wearer's mucosa, tongue, and buccal surfaces, whereas mucormycosis typically involves the maxillary sinus, hard palate, and tissues near the orbit 

Candidiasis is primarily caused by *C. albicans* and multidrug-resistant *Candida auris* [82-85], with risk factors including immunosenescence, removable prostheses with biofilms, and ICU stay. Mucormycosis is caused by *Rhizopus arrhizus* [85], *Mucor* spp. [86], *Lichtheimia* spp. [86], and *Cunninghamella bertholletiae* [87], with uncontrolled diabetes, ketoacidosis, corticosteroid therapy, hyperferritinemia, and immunosuppression as major risk factors such as corticosteroid therapy [88-90].

The pathogenesis of candidiasis involves biofilm formation on mucosa and prostheses, yeast-hyphae switching, and the secretion of proteases and phospholipases [91], thereby affecting the denture-bearing mucosa, tongue, and buccal surfaces. The pathogenesis of mucormycosis features angioinvasion, necrosis due to vascular occlusion, and GRP78-CotH3 receptor interactions, thriving in hyperglycaemic and acidotic conditions [92,93].

Pathogen-specific factors heighten these risks. *C. albicans *remains the predominant isolate in COVID-19 patients, whereas multidrug-resistant *C. auris* is resistant to disinfectants and standard antifungal agents and often forms biofilms on prostheses and hospital surfaces [82-85]. Mucormycosis, conversely, flourishes in hyperglycaemic and acidotic environments, such as uncontrolled diabetes and diabetic ketoacidosis, conditions frequently worsened by corticosteroid therapy for COVID-19 [86,89]. Increased iron levels and impaired neutrophil and macrophage function further enhance the virulence of mucormycosis [90,92].

The clinical impact is considerable. Diagnosis must combine traditional and advanced methods. While clinical examination, blood cultures, histopathology, and imaging remain essential elements [93], innovative techniques such as salivary biomarkers, β-D-glucan assays, matrix-assisted laser desorption/ionization-time of flight (MALDI-TOF), and polymerase chain reaction (PCR)-based diagnostics enable earlier, non-invasive detection [45,86]. Sampling of prosthetic surfaces is essential, as biofilm reservoirs often escape detection by mucosal swabbing [8]. Clinical manifestations include oral thrush, dysphagia, dysgeusia, erythematous or pseudomembranous lesions, and occasional skin pustules in candidiasis [94], whereas mucormycosis presents with facial swelling, sinus congestion, black eschar on the palate or nasal cavity, orbital pain, necrosis, and vision loss [95].

Diagnosis relies on oral examination, blood cultures, β-D-glucan, mannan antigen, PCR, MALDI-TOF, and prosthesis swabs [96,97]. Treatment for candidiasis includes echinocandins, azoles, and strict prosthesis hygiene [98-100], with significant morbidity, malnutrition, and psychosocial distress [101].

Management requires a comprehensive multimodal approach. In mucormycosis, liposomal amphotericin B remains the definitive standard of care, with posaconazole or isavuconazole used as step-down or salvage therapies, often alongside surgical debridement [38,86]. For invasive candidiasis, echinocandins (anidulafungin, micafungin, caspofungin) are considered first-line agents, although oral azoles such as fluconazole and voriconazole remain effective against strains with lower resistance [96,99]. Prosthodontic interventions are essential: removing or thoroughly disinfecting dentures during an active infection is crucial, and experimental microneedle-based antifungal delivery systems may enhance biofilm removal within prosthetic niches [8,48]. Consequently, early detection of candidiasis in denture wearers may serve as an alert for systemic susceptibility to mucormycosis [102,103].

In Table 5, a comparison of candidiasis and mucormycosis is presented.

**Table 5:** Comparative summary of COVID-19–associated candidiasis and mucormycosis in older denture-wearing adults. PCR, Polymerase chain reaction; MALDI-TOF: matrix-assisted laser desorption/ionization time-of-flight; PAS: Periodic acid-Schiff; GMS: Gomori methenamine-silver; qPCR: quantitative PCR.

Feature	Candidiasis	Mucormycosis	
Primary species	*Candida albicans *(most common), *Candida auris* (multidrug resistant) (Arastehfar et al. [82], Chowdhary et al. [83], Chakrabarti and Sood [84])	*Rhizopus arrhizus*, *Mucor* spp., *Lichtheimia* spp., *Cunninghamella bertholletiae* (Ribes et al. [85], Ghosh et al. [86], Chakrabarti and Singh [87])	
Key risk factors	Immunosenescence, removable prostheses with biofilms, ICU stay, corticosteroid therapy (Jerônimo et al. [8], Johansson et al. [1], Riche et al. [88])	Uncontrolled diabetes, ketoacidosis, corticosteroid therapy, hyperferritinemia, immunosuppression (Werthman-Ehrenreich [89], Mehta and Pandey [90], Ghosh et al. [86])	
Pathogenesis	Biofilm formation on mucosa and prostheses; yeast–hyphae switching; secretion of proteases and phospholipases; resistance in *C. auris* (Chakrabarti and Sood [84], Bommanavar et al. [91])	Angioinvasion with hyphal spread; necrosis due to vascular occlusion; GRP78–CotH3 receptor interactions; thrives in hyperglycemic, acidotic states (Honavar [92], Mehta and Pandey [90], Ghosh et al. [86])	
Typical sites	Denture-bearing mucosa, tongue, buccal surfaces (Jerônimo et al. [8], Alhamed et al. [26])	Maxillary sinus, hard palate, orbital tissues, brain (Prabhu and Patel [93], Ghosh et al. [86])	
Clinical signs	Oral thrush, dysphagia, dysgeusia, erythematous or pseudomembranous lesions, skin pustules (Riad et al. [94], Katz et al. [6], Johansson et al. [1])	Facial swelling, sinus congestion, black eschar on palate/nasal cavity, orbital pain, necrosis, vision loss (Werthman-Ehrenreich [89], Gamaletsou et al. [95], Ghosh et al. [86])	
Diagnosis	Oral examination, blood cultures, β-D-glucan, mannan antigen, PCR, MALDI-TOF; prosthesis swabs critical (Pappas et al. [100], Pappas et al. [96], Nieto et al. [97], Jeronimo et al. [8])	Imaging (CT/MRI), tissue biopsy with PAS/GMS stains, PCR/qPCR, histopathology; hallmark = broad aseptate hyphae with 90° branching (Prabhu & Patel [93], Guarner et al. [98])	
Treatment	Echinocandins (first line: caspofungin, micafungin, anidulafungin), azoles (fluconazole, voriconazole), strict prosthesis hygiene/removal (Ben-Ami [99], Pappas et al. [100], Pappas et al. [96])	Liposomal amphotericin B (first line), posaconazole/isavuconazole as step-down or salvage, surgical debridement, adjunct hyperbaric oxygen (Ghosh et al. [86], Ohara et al. [38])	
Prognosis	Significant morbidity; reduced quality of life, malnutrition, psychosocial distress, especially in denture wearers (Johansson et al. [1], Katz et al. [6])	Mortality 18%-56%, worsened by delayed diagnosis and comorbidities; high morbidity in elderly (Ghosh et al. [86])	
Prosthodontic role	Denture hygiene to prevent colonisation; novel strategies such as microneedle antifungal delivery (Peng et al. [48], Jeronimo et al. [8])	Early recognition of oral sentinel lesions; denture management during infection; rehabilitation post-surgical debridement to restore function and QoL (Chouksey et al. [65], Lam et al. [45])	

Although oral candidiasis is less lethal, it significantly affects quality of life by causing dysphagia, dysgeusia, nutritional decline, and psychosocial distress in denture wearers [1,6]. Importantly, prosthodontic rehabilitation not only restores function and appearance but also supports psychological recovery and improves quality of life in post-COVID mucormycosis survivors [65].

Geographic and temporal data indicate that India, the Middle East, and parts of Southeast Asia were disproportionately affected by COVID-19-associated mucormycosis, reflecting high diabetes rates, widespread corticosteroid use, and limited access to preventive oral care [16,86,104]. Peaks in COVID-19 cases coincided with increases in fungal infections, underscoring the need for proactive surveillance, particularly among older adults with dentures.

Critical knowledge gaps remain. Prospective studies are needed to determine whether oral candidiasis predicts progression to mucormycosis, how the oral microbiome changes after COVID, and the effects of standardized denture care, antifungal prophylaxis, and salivary immunomodulation [1,8,9,16]. Collaboration across prosthodontics, geriatrics, and infectious disease is vital to addressing these gaps.

In conclusion, older adults with removable prostheses face disproportionate risks of COVID-19-associated candidiasis and mucormycosis due to systemic, immunological, and prosthetic factors. Early recognition, antifungal therapy, surgical intervention, and meticulous prosthodontic care, supported by preventive strategies such as glucose control, denture disinfection, and antifungal prophylaxis, are essential to reduce morbidity and mortality. Prosthodontic interventions, therefore, go beyond functional restoration to be vital components of infection prevention and comprehensive geriatric rehabilitation post-COVID [65,86].

This narrative review faced several limitations. First, the included studies varied in design, population, and outcome reporting, making direct comparisons or meta-analysis difficult. Many studies relied on small samples, single-center settings, or retrospective data, potentially reducing their generalizability. Case reports and case series made up a significant proportion of the available literature, introducing bias and limiting the strength of causal conclusions. Additionally, inconsistencies in diagnostic criteria for candidiasis and mucormycosis, along with variability in reporting denture use and hygiene practices, may have led to underestimation or misclassification of associations. Geographic bias was apparent, with a disproportionate number of studies from India and the Middle East regions with high diabetes prevalence and CAM incidence, while data from Europe, Africa, and the Americas remain limited. Finally, prospective studies on post-COVID oral health outcomes are scarce, leaving gaps in understanding the long-term effects of denture use in this population.

## Conclusions

This narrative review highlighted that older adults with dentures form a particularly vulnerable group for COVID-19-associated candidiasis and mucormycosis, due to the combination of immunosenescence, systemic comorbidities, and prosthesis-related biofilm development. The combined effect of SARS-CoV-2 immune dysregulation and denture-associated microbial reservoirs increases the risk of infection, leading to significant morbidity and, in the case of mucormycosis, considerable mortality. Prosthodontic rehabilitation, therefore, goes beyond restoring function and aesthetics; it also plays a preventive and therapeutic role in decreasing fungal colonization and supporting recovery.

Future research should focus on long-term studies analyzing the predictive role of oral lesions as early indicators for systemic mucormycosis, alongside interventional trials assessing antifungal prophylaxis, denture disinfection methods, and salivary immunomodulation. Improvements in biomarker-based and molecular diagnostics should be validated in denture-wearing populations to facilitate earlier detection and risk assessment. Collaborative efforts across dentistry, infectious diseases, and geriatrics are essential for developing integrated protocols for high-risk groups. By connecting oral and systemic health care, evidence-based preventive strategies can be created to protect older adults during future pandemics and beyond.
